# Contribution of three-dimensional images in the planning of cementoblastoma resection

**DOI:** 10.1259/bjrcr.20200156

**Published:** 2021-01-08

**Authors:** Stefaan Van Hoe, Eman Shaheen, Karla de Faria Vasconcelos, Joseph Schoenaers, Constantinus Politis, Reinhilde Jacobs

**Affiliations:** 1 Faculty of Medicine, KU Leuven, Leuven, Belgium; 2 OMFS-IMPATH Research Group, Department of Imaging & Pathology, Faculty of Medicine, KU Leuven. Department of Oral and Maxillofacial Surgery, University Hospitals Leuven, Leuven, Belgium; 3 Department of Dental Medicine, Karolinska Institutet, Stockholm, Sweden

## Abstract

Cementoblastomas are rare benign tumours that represent less than 1% of all odontogenic tumours. Complete resection is mandatory to avoid recurrence. This case report describes the contribution of three-dimensional imaging and three-dimensional printing in the pre-operative surgical planning of a large cementoblastoma that not only caused substantial compression on the inferior alveolar and mental nerves, but also caused thinning and partial erosion of the lingual and vestibular cortical bone, thus increasing the risk of pre-operative mandibular fracture.

## Introduction

Cementoblastomas represent less than 1% of all odontogenic tumours.^1–3^ More than 75% occur in the mandible, most often in the molar or premolar region. Cementoblastomas are most common in children and young adults, with 50% occurring before age 20 and 75% occurring before age 30. These tumours are usually associated with an erupted permanent tooth. On two-dimensional (2D) radiographs and CT images, they appear as a periapical, sclerotic, sharply delineated lesions with a low-attenuation halo.^2^ A helpful feature in the differential diagnosis with other sclerotic lesions is that cementoblastomas fuse to the root of the adjacent tooth.^2^ Management of cementoblastomas typically involves complete removal of the tumour and the associated tooth to reduce the likelihood of recurrence.^4^

While 2D radiographs typically allow to diagnose cementoblastoma and other types of lesions based on pattern recognition, cone beam CT (CBCT) images are needed beyond diagnosis, as important assessment tool for planning treatment of several maxillofacial lesions.^5–8^ As compared to 2D panoramic radiography, CBCT provides important additional information such as the buccolingual position of the tumour, and the relationship between the lesion and critical anatomical structures (*e.g.* the inferior alveolar and mental nerve). Three-dimensional (3D) images provide a better assessment of the tumour and its location with respect to adjacent structures, such as teeth and nerves, facilitating more detailed surgical planning. Virtual resection may simulate the post-resection procedure, providing information on the expected post-surgical anatomical situation and foresee potential complications.^9^ Additionally, in cases where the surgical technique and resection margin can be defined pre-operatively, bone graft volumes can be calculated in a reliable way. Moreover, the exact morphology of the bone graft can be assessed using 3D printed models generated from 3D images. Beyond that, in complex cases, titanium plates can be precisely pre-bent on the anatomical model prior to surgery, without time pressure.^9^ Therefore, the literature shows that 3D imaging and 3D printing are of great value for pre-operative surgical planning, reducing not only the duration of surgery but also the stress of the surgeon related to uncertainty of anatomical details and expected course of the procedure.^5–9^

The aim of this case report was to describe the contribution of 3D imaging on the virtual pre-operative planning and treatment of a mandibular cementoblastoma. The contribution of a 3D virtual and printed model to optimise treatment planning and avoid complications is emphasised.

## Case report

A 19-year-old male presented with a swollen left mandible and a palpable mass at the level of element 37. He had mild pain since a few weeks. The symptoms had been attributed to a dental problem by a local dentist, and root canal treatment had been performed, without any effect on the pain or swelling.

**Figure 1.**
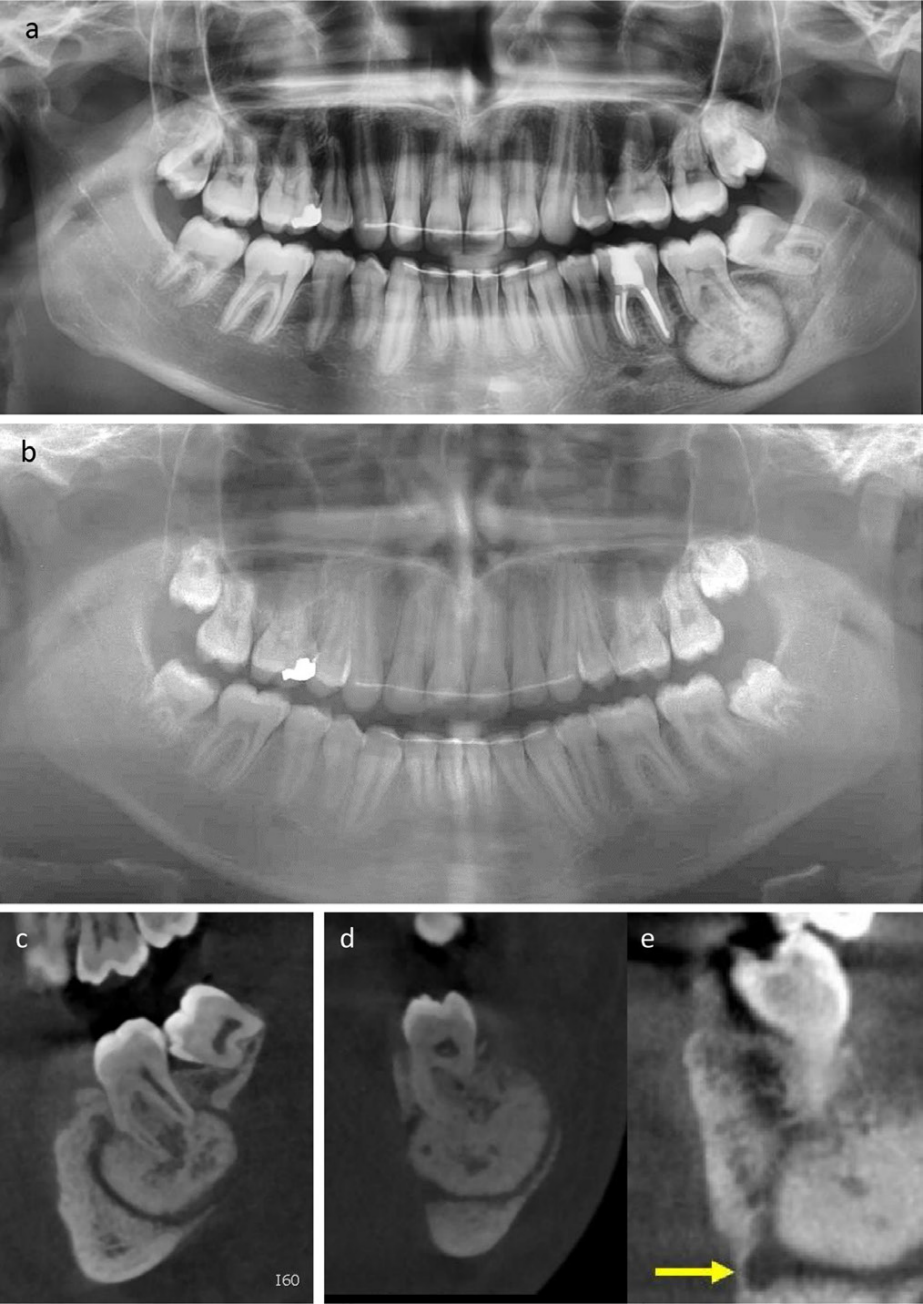
(**a**) Panoramic radiograph showing a sclerotic, sharply delineated lesion with a low- attenuation halo, fused with the root of the adjacent mandibular molar tooth #37. (**b**). Panoramic radiograph taken 3 years earlier for post-orthodontic treatment evaluation showing absence of any mass. (**c–e**). Parasagittal and paracoronal planes, illustrating a heterogeneous sclerotic mandibular mass associated with the element 37 causing cortical thinning and absence of bone both at the lingual and vestibular side (in d). In the enlarged paracoronal image in (**e**), the close relationship of the tumour and the inferior alveolar nerve (arrow) is well seen.

At the initial consultation, a mass was clearly palpable. A panoramic image showed a sclerotic, sharply delineated lesion with a low-attenuation halo, fused with the root of the adjacent molar (Figure 1a). A panoramic radiograph obtained 3 years earlier, for post- orthodontic treatment evaluation, did not shown any tumour sign (Figure 1b).

CBCT showed a heterogeneous mandibular mass associated with the element 37 causing compression and infero-lingual displacement of the inferior alveolar and mental nerve. The mandibular cortex was thinned at the vestibular side and absent at the lingual side (Figure 1c and d). Despite the relatively rapid growth, the diagnosis of benign cementoblastoma was proposed based on the classic appearance as a periapical, sclerotic, sharply delineated lesion with a low-attenuation halo, directly fused to the root of the tooth.

**Figure 2.**
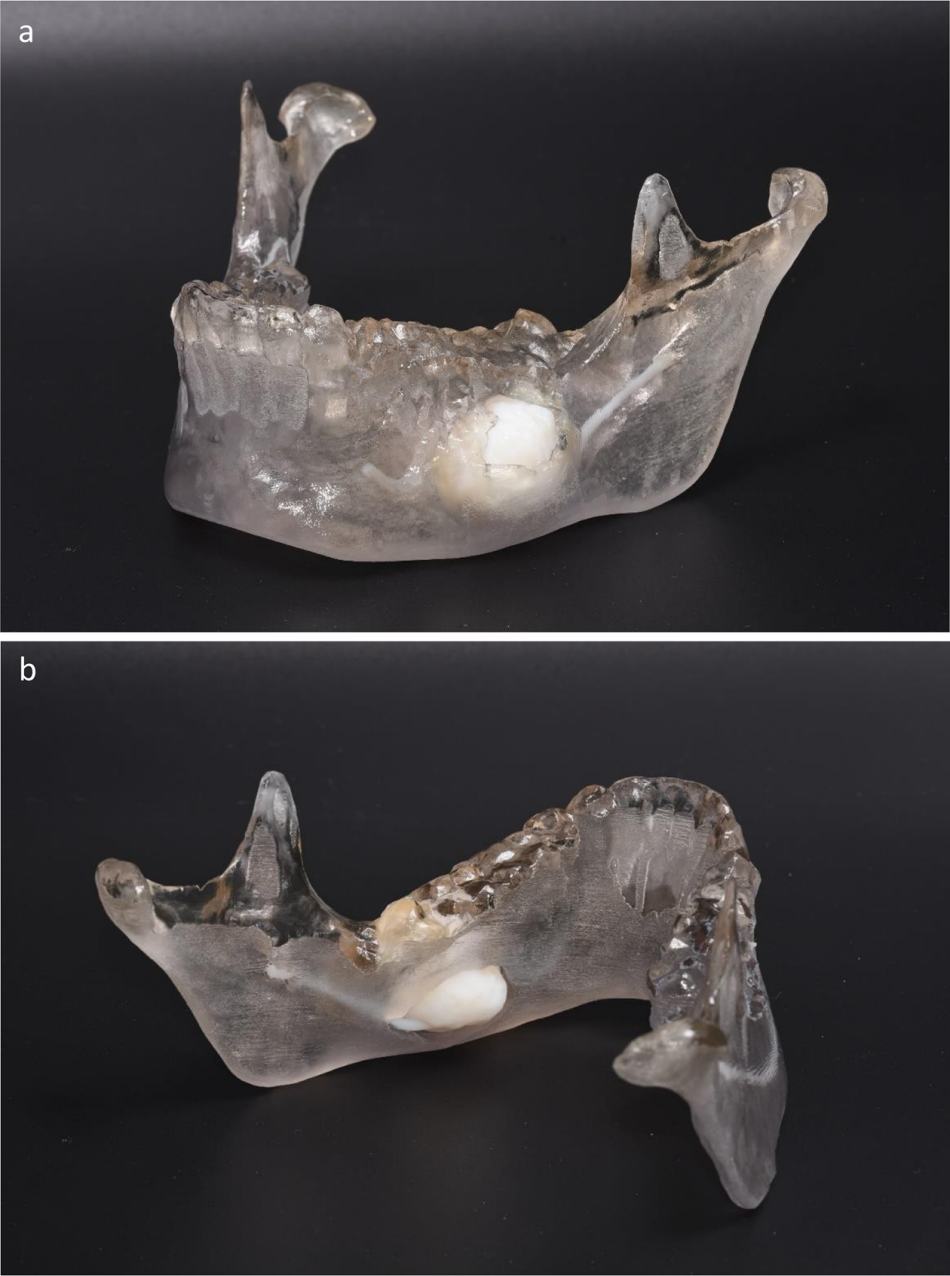
(**a, b**). Three-dimensional model showing the affected anatomic area, with intimate relation of the tumour and the inferior alveolar and mental nerves. The absence of cortical bone at the vestibular side is well depicted in (**a**) and the absence of cortical bone at the lingual side is well seen in (**b**).

3D renderings showing the affected anatomic area were obtained (Figure 2a and b). Considering lesion size and location, a possible pathological fracture of the mandible pre- or post-operatively was considered.

In order to prepare for such risk or complication, 3D planning and printing were made available. The virtual 3D planning was performed in PROPLAN software (Materialise, Leuven, Belgium). The pre-operative CT patient Digital Imaging and Communications in Medicine (DICOM) images were imported into the software where threshold was applied to segment the mandible and the lesion. Both objects were further manually refined and 3D models were created. The inferior alveolar mandibular nerves were traced and 3D models were created. All 3D models were revised by the surgeon prior to export. These models were then exported as Standard Template Library (STL) files for 3D printing. The STL files were imported into the software of the professional Objet Connex 350 printer (Stratasys, Eden Prairie, MN) which is a polyjet printer with layer thickness of 30 µm. The mandible was printed in transparent hard material while the lesion and nerves in hard white material.^10,11^

The 3D printed model allowed for possible fast and accurate (pre)bending of an osteosynthesis plate. Furthermore, considering the tumour size as well as the size of the expected post-surgical bone defect, the possibility for needing a bone graft was also taken into account.

**Figure 3.**
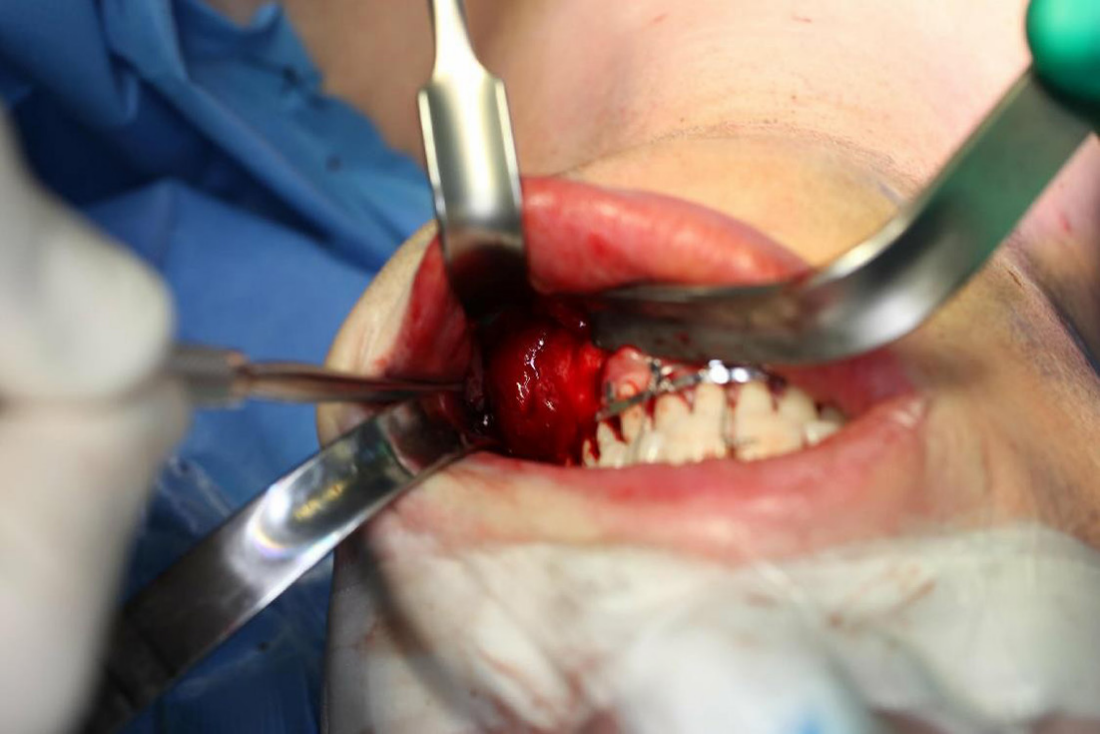
Intraoperative picture.

**Figure 4.**
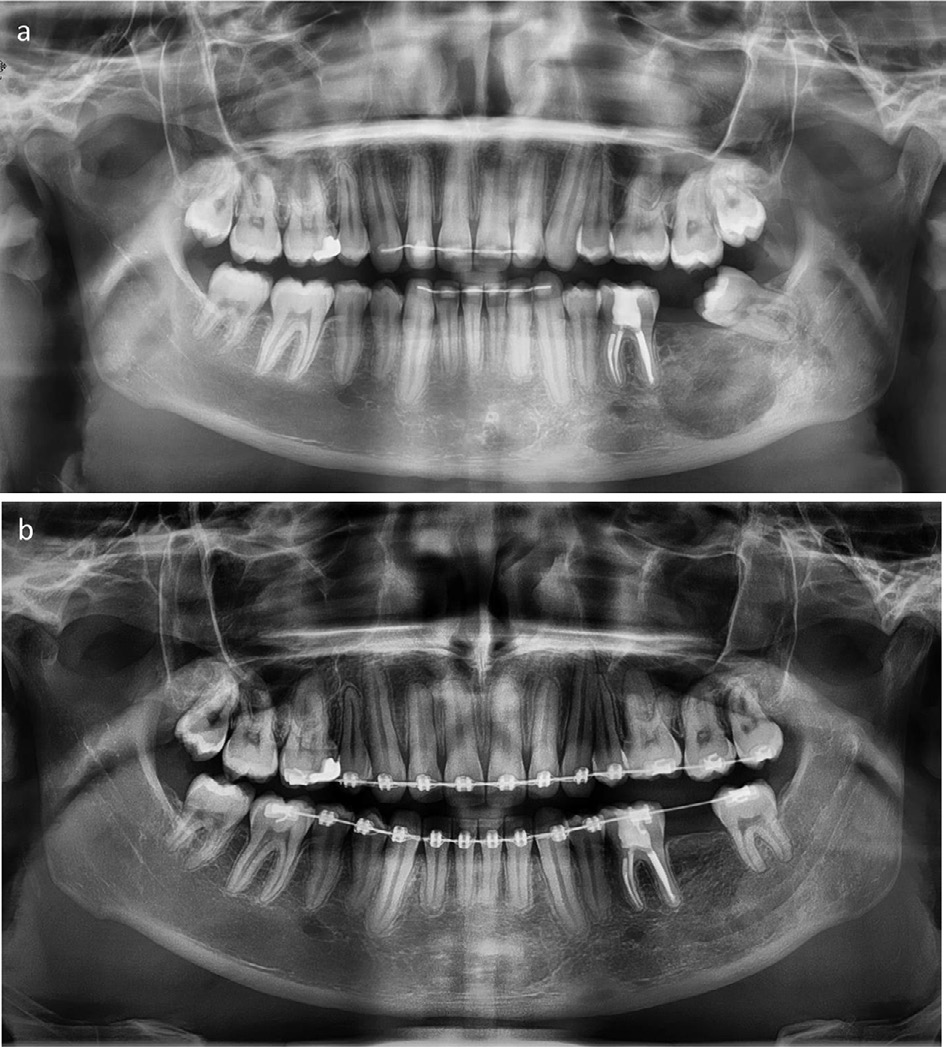
(**a, b**). Panoramic radiographs obtained 6 months after surgery (**a**), showing normal healing process in progress, and 4 years after surgery (**b**), showing complete healing, without recurrence. In (**b**), the position of the wisdom tooth adjacent to the resection area has been corrected successfully after new orthodontic treatment.

A standard surgical approach was used (Figure 3). The tumour could be completely dissected, and the mental nerve could be separated from the tumour without injuring the nerve. Element 37 was removed. The cementoblastoma was split in pieces using a surgical drill, and the pieces were carefully removed, taking care to avoid mandibular fracture or nerve injury. The inferior alveolar nerve could be identified at the bottom of the resection cavity and appeared intact. The mandibular wisdom tooth was not removed in order to minimise the risk of pre- and post-operative fracture. While the mandibular cortex was deficient at the lingual side (as shown pre-operatively by the CBCT images and 3D model), the lower part of the mandibular bone could be kept intact during the procedure and fracture did not occur. The resection cavity was filled with 8 L-PRF membranes. Tight intermaxillary fixation was applied for 6 weeks. No bone grafts were placed in order to facilitate interpretation of the post-operative evolution, taking into account the possibility of tumour recurrence. The post-operative course was uneventful. Follow-up imaging obtained 4 years and 6 months after surgery showed normal healing with progressive new bone formation and closure of the bone defect (Figure 4a and b).

## Discussion

Recent articles have focused on the contribution of 3D images in pre-operative assessment of maxillofacial tumours and other abnormalities.^5–9^ In this case report, we described how CBCT images were needed beyond diagnosis for pre-operative planning and pre-operative support in a patient with cementoblastoma.

While cementoblastomas are benign and usually slowly growing, relatively rapid growth was observed in our case, with a completely negative panoramic radiograph 3 years before.

The case described here was challenging for the surgeon because of two reasons: first, the mass was in close contact with the inferior alveolar and mental nerves, indicating a risk for peroperative nerve damage. Second, because of the extensive thinning (and even absence) of the surrounding mandibular bone, the risk of fracture during and post-surgery was substantial.

This case report showed that 3D imaging and printing played an important role in the pre-operative surgical planning for cementoblastoma removal. As described above, it allowed the surgical team to optimally assess the two major pre-operative risks in this case, *i.e.* damage of the inferior alveolar nerve and unforeseen peroperative fracture. Because of the clear visualisation of the relation of the tumour to the adjacent nerves on the 3D images, the surgeon could perform a meticulous resection while sparing the nerve. Similarly, the 3D model allowed to assess the risk of pre-operative fracture, and even more important, to be prepared for a plan of action in case such as fracture would occur inadvertently.

## Learning points

- 3D imaging and 3D printing can help in the pre-operative surgical planning of cementoblastomas.
- Cementoblastomas can be diagnosed with panoramic views, however, this 2D image modalities do not provide all the necessary information to perform a safe surgical procedure in case of extensive lesions.
